# Complementarity determining regions and frameworks contribute to the disulfide bond independent folding of intrinsically stable scFv

**DOI:** 10.1371/journal.pone.0189964

**Published:** 2017-12-18

**Authors:** Anna Gąciarz, Lloyd W. Ruddock

**Affiliations:** Faculty of Biochemistry and Molecular Medicine, University of Oulu, Oulu, Finland; New England Biolabs Inc, UNITED STATES

## Abstract

CyDisCo is a system facilitating disulfide bond formation in recombinant proteins in the cytoplasm of *Escherichia coli*. Previously we screened for soluble expression of single chain antibody fragments (scFv) in the cytoplasm of *E*. *coli* in the presence and absence of CyDisCo, with >90% being solubly expressed. Two scFv, those derived from natalizumab and trastuzumab, were solubly produced in high amounts even in the absence of folding catalysts i.e. disulfide bond formation is not critical for their folding.

Here we investigate the contribution of the framework and the complementarity determining regions (CDRs) of scFv to the disulfide-independence of folding. We swapped CDRs between four scFv that have different properties, including two scFv that can efficiently fold independently from disulfide bonds and two more disulfide-dependent scFv. To confirm disulfide-independence we generated cysteine to alanine mutants of the disulfide-independent scFv. All of the scFv were tested for soluble expression in the cytoplasm of *E*. *coli* in the presence and absence of the oxidative folding catalysts Erv1p and PDI.

Eight of the hybrid scFv were solubly produced in the presence of CyDisCo, while seven were solubly produced in the absence of CyDisCo, though the yields were often much lower when CyDisCo was absent. Soluble expression was also observed for scFv natalizumab and trastuzumab containing no cysteines. We compared yields, thermal stability and secondary structure of solubly produced scFv and undertook binding studies by western blotting, dot blotting or surface plasmon resonance of those produced in good yields. Our results indicate that both the CDRs and the framework contribute to the disulfide-dependence of soluble production of scFv, with the CDRs having the largest effect. In addition, there was no correlation between thermal stability and disulfide-dependence of folding and only a weak correlation between the yield of protein and the thermal stability of the protein.

## Introduction

Antibodies are tetrameric proteins consisting of two heavy and two light chains that are held together by inter-chain disulfide bonds. The light chain comprises a variable (V_L_) and a constant domain (C_L_), while the heavy chain of IgG_1_ antibody subtype consists of one variable domain (V_H_) and three constant domains (C_H_1, C_H_2, C_H_3). Each domain is stabilized by one disulfide bond. The variable domains each have three hypervariable loops, known as the complementarity determining regions (CDRs), which are the main regions engaged in antigen binding. The CDRs are supported by the framework region which determines the structure of the variable domain.

Variable fragments (Fv) are the smallest fragments of an antibody that can bind to the antigen with similar affinity and specificity of full length antibody. Non-covalently associated functional Fv fragments were produced in the periplasm of *E*. *coli* already in 1988 [1]. However, the non-covalent re-association of Fv fragments proved unstable, therefore Bird et al circumvented this obstacle by linking the V_H_ domain to the V_L_ domain through a short flexible peptide, generating a single chain Fv or scFv, so that both domains could be expressed from one gene and provide equimolar expression of both Fv [2]. Each Fv contains an intra-domain disulfide bond, therefore scFv expression usually requires an oxidizing environment such as found in the eukaryotic endoplasmic reticulum or bacterial periplasm. The disulfide bond of each Fv domain is highly conserved and critical for domain stability and solubility [3,4]. The overall stability of an antibody or an antibody fragment depends not only on intrinsic stability of each domain, but also on the stability of domain interfaces [5,6]. Only intrinsically very stable scFv can fold in the absence of both disulfide bonds [7] and in reducing environments, such as found in the cytoplasm of a cell, most scFv will form non-functional insoluble aggregates.

Although antibodies are secreted proteins there is increasing interest in intrabodies i.e. antibodies or antibody fragments that can be expressed and retained intracellularly. Targeting an intra-body to the cell enables the study of protein function *in vivo* or the modulation of molecular events inside the cell, e.g. stabilization of protein–protein interactions, neutralization of intracellular antigens or even catalyzing reactions [8–10]. The potential intrabody needs to be hyperstable to fold in the reducing environment of the cytoplasm.

So far only a few scFv have been reported to be soluble and functional in the absence of both disulfide bonds. The seminal works in the field towards intrabody production were circa 20 years ago. i) Ohage and Steipe showed that by rational engineering it was possible to construct hyperstable V_L_ domains which were able to fold in the cytoplasm [11]; ii) Proba *et al* by means of molecular evolution (DNA shuffling and phage display) generated stable and functional scFv lacking disulfide bonds in both V_H_ and V_L_ [12] based on the scFv fragment of the levan binding antibody ABPC48, which naturally misses one of the conserved cysteine residues in V_H_ [13]; iii) Martineau *et al* through random genetic mutation, screening and selection generated high yields of disulfide free scFv against β-galactosidase in the cytoplasm of *E*. *coli* [14]; iv) Tavladoraki *et al* showed that scFv derived from the anti-viral antibody F8 was functionally expressed in the cytoplasm of a transgenic plant and *E*. *coli*, and had free sulfhydryl groups [8].

The cysteines involved in disulfide bonds are located in the scFv framework which could imply that these regions are responsible for disulfide-dependency of scFv folding and stability. It has been reported that intrinsically stable scFv could be used as a scaffold for grafting antigen binding loops from other antibodies to generate more soluble and stable scFv [15,16]. Grafting of antigen binding regions was first applied for antibody humanization, in order to decrease the risk of immune response triggered by a rodent antibody [17]. Carter *et al* humanized anti-HER2 murine antibody (mumAb4D5) and generated an antibody with a very favourable folding properties [18]: Jung and Plückthun used the framework of anti-HER2 scFv (under the name “4D5”) for grafting CDRs of aggregation prone fluorescein binding antibody 4-4-20 and the resulting scFv showed improved solubility and thermal stability [15]. Anti-HER2 scFv framework also served as scaffold for transplantation of CDRs from EGP-2-binding MOC31 scFv and the generated scFv showed increased stability and better expression [16]. Moreover, Wörn and Plückthun generated a biologically functional cysteine-free derivative of anti-HER2 scFv by mutating cysteine residues to valine or alanine; however this scFv variant formed inclusion bodies in the periplasm of *E*. *coli* and *in vitro* refolding was necessary to obtain active protein (7).

While transplanting hypervariable loops has proved to be sufficient to preserve the antigen binding affinity [19,20], in many cases it has been necessary to transfer also several framework residues from the paternal scFv to ensure retention of the binding properties: these framework residues were shown have crucial influence on the conformation of particular CDR or even to be directly involved in antigen binding [21–24].

It has been known for nearly 20 years that some scFv antibody fragments are able to fold in the absence of disulfide bonds [8,12,13]. The disulfide-dependence/independence of folding must be linked to the stability of the native-like fold with no disulfide bond [e.g. 11] but it is unclear the degree to which this is dependent on the framework region of the scFv or the CDRs. Since the CDRs are in the core of the framework and are not in contact with the disulfide bond in either the V_L_ or V_H_ domain [25], the simplest hypothesis is that the framework is more essential than the CDRs for disulfide-dependence/independence of folding and if this is true then it should be possible to swap CDRs from disulfide-dependent scFv into disulfide-independent frameworks and obtain folded protein in the absence of disulfide formation, but not *vice versa*. To test this hypothesis a rapid screen is required and one which does not introduce a bias due to different compartments being used or due to the sole use of cysteine mutants in the scFv. It has been reported that folding for disulfide-independent scFv is limited by prolyl isomerization [4,26] and so the presence of sufficient levels of catalysts for this slow folding step is essential. Previously [27] we reported soluble expression of variety of scFv and Fabs in the cytoplasm of *E*. *coli*, in the presence of folding catalysts, a sulphydryl oxidase Erv1p and disulfide bond isomerase, PDI, that form a system called CyDisCo (cytoplasmic formation of disulfide bonds in *E*. *coli*). We tested expression of fragments of different antibody isotypes (IgG, IgM, IgA, IgE), subclasses (IgG_1_, IgG_2_, IgG_4_, IgA_1_) and derived from different species (human, murine, humanized). Out of eleven tested scFv soluble expression was observed for ten. Yields varied from 4 mg/L up to 271 mg/L with no correlation between the expression level and any specific antibody type, with the most abundant and the most poorly expressed being IgG_1_. Each scFv expression was tested in parallel in the absence of folding helpers and only two scFv, natalizumab and trastuzumab (in [27] designated as “Tysabri” and “Herceptin”, respectively), were efficiently expressed solubly in the absence of catalysts of disulfide bond formation. The trastuzumab derived scFv has identical V_H_ and V_L_ domains as the anti-HER2 scFv used by Jung and Plückthun [7]. When using CyDisCo the six cytoplasmic *E*. *coli* peptidyl-prolyl cis-trans isomerases are present and hence prolyl isomerization should be efficiently catalyzed. Furthermore parallel +/- CyDisCo screening for production is facile in any *E*. *coli* strain and no physiological changes in response to CyDisCo have been reported. Hence CyDisCo could be used to screen for disulfide-dependency of folding of scFv.

Here we examine the contribution of CDRs and framework regions to the disulfide-dependency of folding of scFv. Four scFv were selected for the study, two that showed a high degree of disulfide-independence (trastuzumab and natalizumab) and two more disulfide-dependent (IgA_1_ and Maa48). We swapped CDRs and tested for disulfide-dependence of folding in the cytoplasm of *E*. *coli* using the CyDisCo system. To verify disulfide-independent folding we mutated all four cysteines to alanine in the trastuzumab and natalizumab scFv. We also studied the thermal stability and secondary structure of solubly expressed scFv to examine for correlations with disulfide-dependency. While not directly relevant to the question of disulfide-dependence of folding we also undertook binding studies by western blotting or dot blotting or surface plasmon resonance of most of the scFv produced to aid other researchers in the field of hybrid scFv production.

## Materials and methods

### Strains and expression vectors

All molecular biology performed in this study used the *E*. *coli* strain XL1 Blue (*recA1 endA1 gyrA96 thi-1 hsdR17 supE44 relA1 lac F’[proAB lacI lacZΔM15 Tn10 Tet*^*r*^*]*; Agilent Technologies), while all protein production was performed in the Keio’s collection parental strain BW25113 *(rrnB3 ΔlacZ4787 hsdR514 Δ(araBAD)567 Δ(rhaBAD)*568 *rph-1*) [28,29].

Expression vectors (Table 1) were made by standard molecular biology techniques. The scFv expression vector used was based on pET23 in which the T7 promoter was replaced with *tac* promoter, as described previously [27]. All vectors encoded V_L_ and V_H_ in a V_L_-(Gly_4_Ser)_3_-V_H_-GSH_6_ format.

**Table 1:** Plasmids used in this study.

Construct	Plasmid	Selection*	Reference
CyDisCo components: coErv1p and coPDI	pMJS205	Cm^R^	[27]
CyDisCo negative control	pAG82	Cm^R^	[27]
HER2 V529-P625, C-terminal GSH_6_ tag	pFH3	Amp^R^	This study
**scFv (type)**			
trastuzumab (IgG_1_ humanized)	pJV78	Amp^R^	[27] (as “Herceptin scFv”)
trastuzumab, C24A, C89A, C149A, C223A	pAG158	Amp^R^	This study
natalizumab (IgG_4_ humanized)	pJV81	Amp^R^	[27] (as “Tysabri scFv”)
natalizumab, C24A, C89A, C148A, C222A	pAG159	Amp^R^	This study
IgA_1_ (human)	pJV84	Amp^R^	[27]
Maa48 (IgG_1_ human)	pJV79	Amp^R^	[27]
**scFv with grafted CDRs (framework-CDRs)**			
trastuzumab-natalizumab	pAG156	Amp^R^	This study
trastuzumab-IgA_1_	pAG160	Amp^R^	This study
trastuzumab-Maa48	pAG111	Amp^R^	This study
natalizumab-trastuzumab	pAG157	Amp^R^	This study
natalizumab-IgA_1_	pAG114	Amp^R^	This study
natalizumab-Maa48	pAG113	Amp^R^	This study
IgA_1_-trastuzumab	pAG117	Amp^R^	This study
IgA_1_-natalizumab	pAG155	Amp^R^	This study
Maa48-trastuzumab	pAG115	Amp^R^	This study
Maa48-natalizumab	pAG116	Amp^R^	This study

* Selection markers are ampicillin (Amp^R^) or chloramphenicol (Cm^R^)

The trastuzumab, natalizumab, IgA_1_ and Maa48 scFv were constructed and described previously (24). ScFv with swapped CDRs along with trastuzumab and natalizumab scFv with all cysteines mutated to alanine were synthetized by GenScript and cloned Nde I / BamH I into an expression vector in frame with a C-terminal GSH_6_-tag. For scFv with swapped CDRs the framework residue 71 of both the V_H_ and V_L_ domains of the hybrid were matched with the corresponding residue of the CDR donor as these framework residues have been implicated in preserving the structure and function of the CDRs [24, 30, 31].

CyDisCo components Erv1p and PDI were co-expressed from vector pMJS205; pAG82 plasmid was used as the negative control, since it shares the same backbone as pMJS205, but does not carry any recombinant protein genes. The construction of pMJS205 and pAG82 was described previously [27].

A codon optimized synthetic gene of the V529-P625 fragment of trastuzumab’s antigen HER2 was ordered from GenScript and cloned Nde I/ BamH I into an expression vector in frame with a C-terminal GSH_6_-tag.

All genes used were codon optimized for expression in *E*. *coli* and all constructs were sequenced to ensure absence of errors in the cloned genes.

### Protein expression

*E*. *coli* BW25113 strains transformed with a vector for a scFv expression along with either the CyDisCo vector for cytoplasmic disulfide bond formation or a control vector were restreaked twice from single colonies (to minimize the effects of clonal variation) before being stored as glycerol stocks at -70°C. From these stocks they were streaked onto LB-agar plates containing 100 μg/mL of ampicillin and 35 μg/mL of chloramphenicol and incubated overnight at 37°C for expression in EnPresso B media (Biosilta Ltd) according to the manufacturer’s instructions. In brief, the next day 2 mL LB media containing 2 g/L of glucose was seeded with a few colonies from the LB-agar plate and incubated at 30°C, 200 rpm for 7–8 h. The pre-cultures were used to inoculate the EnPresso B media containing suitable antibiotics and incubated at 30°C, 250 rpm overnight. The next morning the cultures were induced with 0.5 mM IPTG and boosted according to the manufacturer’s instructions. The cultures were harvested 24 h after induction by centrifugation at 3 220 x g for 20 min at 4°C, the pellets were resuspended to the initial culture volume in 50 mM sodium phosphate buffer, pH = 7.4, containing 20 μg/mL of DNase (Roche) and 0.1 mg/mL of hen egg lysozyme (Sigma Aldrich), incubated at room temperature for 15 min to allow the lysozyme to act and then frozen at -20°C. No toxic effects due to recombinant scFv expression were observed.

The initial expression screen was done in 3 mL cultures of EnPressoB media (BioSilta) grown in 24-deep well plates, at 250 rpm in an incubator with a 5 cm radius of gyration. Cultures of strains that produced visible amounts of purified scFv in the initial screen were scaled up to 25 or 50 mL of EnPressoB in shake flasks and cultivated at 250 rpm in an incubator with a 2.5 cm radius of gyration. Oxygen permeable membranes were used for all cultures as Erv1p uses molecular oxygen to generate disulfide bonds.

Similarly, the HER2 fragment was expressed along with CyDisCo components in BW25113 in 50 mL cultures of EnPressoB media.

### Protein purification

All constructs expressed had a hexa-histidine tag at their C-terminus to allow purification with standard immobilized metal affinity chromatography (IMAC). The resin used was HisPur Cobalt Superflow Agarose resin (Thermo Scientific). The cells were lysed by one freeze-thawing cycle, as this prevents scFv aggregation and degradation on repeated freeze-thawing. This protocol results in no/minimal differences in intensity in bands for soluble proteins in total vs soluble lysate analysis by Coomassie stained SDS-PAGE implying complete or near complete lysis. Thawed lysates were centrifuged at 3 220 x g at 4°C for 20 min and the supernatants loaded onto gravity-flow columns containing 0.5 mL (3 mL lysates) or 1 mL (12.5 mL lysates from either 25 mL or 50 mL cultures) of HisPur Cobalt Superflow Agarose IMAC resin (Thermo Scientific) which had been pre-equilibrated in 50 mM phosphate buffer, pH = 7.5. The soluble lysates were incubated on the resin for 5 min, drained, the resin washed with 5 mL of equilibration buffer and then with 4 x 5 mL of wash buffer (0.3 M NaCl, 15 mM imidazole and 50 mM sodium phosphate buffer, pH = 7.5), followed by 5mL of equilibration buffer. The protein was eluted with 50 mM EDTA, 50 mM sodium phosphate buffer, pH = 7.4); 2.1 mL of buffer was used to elute from 0.5 mL of resin and 4 mL of elution buffer to elute from 1 mL of resin. 2.5 mL of protein was desalted into phosphate buffered saline (137 mM NaCl, 2.7 mM KCl, 20 mM sodium phosphate buffer, pH = 7.4) using a PD-10 columns (GE Healthcare) and stored at 4°C or -20°C. Using this purification method the scFv contained minor amounts of *E*.*coli* proteins. Since the scFv all have different biophysical properties (Table 2) there was not a single second purification step which could be used consistently for all and we were concerned that using different methods might in some way bias the results. We were also concerned that the scFv produced without CyDisCo would undergo *ex vivo* air oxidation and that this would increase with the number of purification steps—with the degree being effected by purification conditions such as pH.

**Table 2:** Biophysical properties of the scFv purified in this study.

scFv	CyDisCo	Expected mass (Da)	Experimental mass (Da) − NEM	Experimental mass (Da) + NEM	pI
trastuzumab	-	27189	27188	27688	8.6
	+	27185	27185	27185	
trastuzumab, CA	-	27061	27060	27060	9.0
	+	27061	27060	27060	
natalizumab	-	27755	27753	28256	7.7
	+	27751	27751	27752	
natalizumab, CA	-	27627	27626	27626	7.9
	+	27627	27626	27626	
IgA_1_	-	27346	27342	27342	8.7
	+	27342	27342	27342	
Maa48	+	26336	26336	26337	8.8
**scFv with grafted CDRs** **(framework-CDRs)**					
trastuzumab-natalizumab	-	27650	27649, 27648, 27647	27896, 28021, 27771, 28146	8.5
	+	27646	27646	27646	
trastuzumab-IgA_1_	-	27528	27527	27523, 27775, 27901	9.3
	+	27524	27523	27523	
trastuzumab-Maa48	+	26787	26786	26786	9.2
natalizumab-trastuzumab	-	27294	27289	27289	7.7
	+	27290	27289	27291	
natalizumab-IgA_1_	-	27633	27629	27629	9.1
	+	27629	27628	27628	
IgA_1_-natalizumab	-	27469	27465	27464	6.7
	+	27465	27465	27464	
Maa48-trastuzumab	+	26735	26735	26734	7.1
Maa48-natalizumab	-	27199	27196	27195	7.1
	+	27195	27195	27195	

scFv frameworks which have all four cysteines mutated to alanine are indicated as CA.

HER2 fragment was purified on IMAC and desalted on PD-10 column in the same way as scFv. After that it was additionally purified on 6 mL Resource Q anion exchange column (GE Healthcare) using an ÄKTA-FPLC system (Amersham Pharmacia biotech) operated via UNICORN Control Software. The protein was diluted two times in 20 mM sodium phosphate buffer, pH = 7.4 and loaded onto the column. The protein was eluted with linear gradient of 0 to 1M NaCl in 20 mM BASE, pH = 7.4. 1 mL elution fractions were collected and analyzed on SDS-PAGE gel. The fractions containing pure HER2 fragment were combined and used for further analysis.

### Biophysical characterization

Protein concentration was determined by measurement of absorbance at 280 nm. From each measured value A280 = 0.032 was subtracted corresponding to the absorbance of contaminating *E*. *coli* proteins that co-purified on IMAC and that were observed as weak bands on SDS-PAGE even in samples not yielding any scFv. For most scFv this correction was less than 2% of the A280. Protein concentration was calculated using molar extinction coefficients based on amino acid composition.

Molecular masses were measured by Liquid chromatography mass spectrometry (LCMS) with an Aquity UPLC-system (Waters) connected to a Synapt G1 Q-ToF—type mass spectrometer (Table 2). The analytical column was a BEH 300 C4, 2.1 x 100 mm (Waters) run at 0.4 mL/min using a gradient from 3% acetonitrile in water/0.1% FA to 43% acetonitrile over 10 min. Samples were acidified with trifluoroacetic acid to about 0.5% v/v and 2–5 μL of sample was injected. The mass spectrometer was operated in sensitivity mode with lock mass corrected 1 sec scans in continuous mode for m/z 100 to 2000. Capillary voltage was 3.5 kV, cone voltage 30 V. Mass spectra were base line subtracted and deconvoluted with MaxEnt1. To examine the free thiol status of the proteins samples were treated with 25 mM N-ethyl malemide (NEM) for 10 min prior to analysis by LCMS. NEM adducts add 125 Da to free thiol groups i.e. 250 Da per unformed disulfide in the scFv. For one scFv a small amount (circa 3%) non-specific labelling by NEM was observed.

Far-UV circular dichroism spectra were recorded on a Chirascan-Plus CD spectrophotometer. All scans were collected at 22°C as an average of four scans, using a cell with a path length of 0.1 cm, scan speed 1 nm/s, step size 1 nm, a spectral band width of 1 nm. The HT voltage did not exceed 750V.

Thermal shift assay or thermofluor assay was performed using 7500 Real Time PCR System (Applied Biosystems) with v2.0.5 software. The total reaction volume per well was 25 μL. After optimization 8μM of protein was chosen to be used as giving the best signal:noise ratio. For some low yield scFv 2μM (trastuzumab framework—IgA1 CDR hybrid and IgA1 framework-natalizumab CDR hybrid) or 0.5μM (Maa48) were used instead. Tests were undertaken to ensure that the melting temperatures obtained was not concentration dependent over this range. For the assay 22.5 μL of 0.5–8 μM of scFv in 50 mM phosphate buffer, pH = 7.5, was mixed with 2.5 μL of 50 x SYPRO Orange Protein Gel Stain (Sigma Aldrich)-dye (original stock 5000x, diluted to 50x prior the use in 50 mM phosphate buffer, pH = 7.5) and loaded on to a 96-well plate (Micro Amp reaction plate, Applied Biosystems) in 3–6 replicates. Phosphate buffer with dye was used as a negative control. The plate was sealed with heat resistant adhesive film. The samples were heated in the PCR System from 21 to 90°C, in increments of 0.75°C/min and the fluorescence measured. The melting temperature (T_m_) was determined by examining the derivative of the fluorescence with time.

### Activity studies

Binding of scFv with trastuzumab’s CDRs to HER2 [32] was analyzed via two methods: standard western blotting and surface plasmon resonance (SPR). For western blotting 250 ng of recombinant HER2/Erb2 protein manufactured by Sino Biological (extracellular region T23-T652 with a DDDDK-tag at the C-terminus) was mixed with non-reducing SDS-PAGE sample buffer, heated for 5 min at 95°C and loaded on a 12.5% SDS-PAGE gel together with 150 ng of corresponding scFv as control for anti-His-tag binding. The protein was transferred to the Immobilon-P (PVDF) membrane in vertical electrophoresis apparatus at 100 V, 1.5 h, at room temperature, in transfer buffer (190 mM glycine, 25mM Tris, 10% ethanol). The membrane was incubated shaking in 5% milk solution in Tris-buffered saline (TBS; 20 mM Tris, 137 mM NaCl, pH = 7.5) overnight at 4°C. The blot was rinsed three times in TBS buffer containing 0.1% Tween 20 (TBS-T). IMAC purified and desalted scFv was diluted to 0.3 μg/mL in TBS buffer, added to the membrane, and incubated shaking for 1 h at room temperature. The blot was washed three times in TBS-T buffer. HRP-labelled anti-His tag antibody (GenScript) was diluted to 1:5000 in TBS buffer and the membrane incubated with this antibody for 1 h at room temperature. The membrane was washed three times with TBS-T and incubated with substrate for HRP (Pierce ECL Western Blotting Substrate) for 1 min. The chemiluminescent signal was detected with ChemiDoc Imaging System (Bio-Rad). Since the commercial HER2 was found to be heterogeneous, we produced using CyDisCo a HER2 fragment V529-P625 that contains the non-linear epitope to which trastuzumab binds (1N8Z; [33]). A Biacore T200 SPR system was then used to study the interaction of scFv containing trastuzumab’s CDRs with HER2 fragment. HER2 fragment eluted from the final anion exchange purification step was biotinylated using EZ-Link NHS-PEG_4_-Biotin (ThermoScientific) according to the manufacturer’s instructions. In brief, the HER2 fragment was incubated for 30 min at room temperature with 20x molar excess of biotinylation reagent. The extra biotin was removed from the protein solution on a PD-10 column and biotinylated protein was concentrated on Amicon Ultra -0.5 mL Centrifugal Filter (-3K). HBS-EP (10 mM HEPES, 150 mM NaCl, 3 mM EDTA, 0.00005% v/v P20) was used as the running buffer for the immobilization of biotinylated HER2 on a carboxymethylated dextran chip pre-immobilized with streptavidin (Series S sensor chip SA, GE Healthcare). The sensor surface was conditioned with 1 M NaCl in 50 mM NaOH prior to ligand immobilization. For binding of the scFv to the HER2 fragment 50 mM sodium phosphate pH 7.4 was used as running buffer. An ionic cocktail (0.46 M KSCN, 0.92 M urea, 1.83 M MgCl2, 1.83 M guanidine hydrochloride) was applied for the regeneration of the interaction surface. Sequential dilutions of each scFv in the range 0.2–5 μM were prepared in 50 mM sodium phosphate, pH = 7.4, on a 96-well plate. Each sample was analyzed in triplicate.

Binding of scFv containing natalizumab’s CDRs was tested via dot blotting. Full length human ITGA4 (antigen CD49, α4 subunit of VLA-4 receptor; OriGene Technologies (or Abnova (data not shown))) was used as the antigen [34] and ITGA4 fragment R92-G201 (Abnova) as a negative control. Immobilon-P membrane was activated in methanol, rinsed with water and air dried for few minutes. 80 ng of ITGA4 full length or ITGA4 fragment and 200 ng of scFv as a control for secondary antibody binding were loaded onto the membrane and air dried for about 30 min. The membrane was incubated shaking in 5% milk solution in TBS, rinsed in TBS buffer and incubated for 1 h at room temperature with 6 μg/mL of purified and desalted scFv diluted in TBS buffer. The blot was washed, incubated with HRP-labelled anti-His Tag Antibody (GenScript), washed, incubated with HRP substrate and the chemiluminescent signal was detected in the same way as for anti-HER2 western blots above.

The IgA_1_ antibody used in this study binds specifically to the C-terminus of β-tubulin [35–37]. A Biacore T200 SPR system was used to study the interaction of scFv containing the CDRs from the IgA_1_ with a synthetic peptide DATAEEEEDFGEEA corresponding to residues 427–440 of human β-tubulin (NCBI Reference NP_821133.1) immobilized on the chip. The peptide was synthetized by Fmoc chemistry on an ABI433A Peptide Synthesizer (Applied Biosystems). The peptide was biotinylated at the C-terminus and immobilized on carboxymethylated dextran chip pre-immobilized with streptavidin (Series S sensor chip SA, GE Healthcare). For immobilization HBS-EP (10 mM HEPES, 150 mM NaCl, 3 mM EDTA, 0.00005% v/v P20) was used as the running buffer. The sensor surface was conditioned with 1 M NaCl in 50 mM NaOH prior to ligand immobilization. The system was washed with 50% isopropanol in 1 M NaCl and 50 mM NaOH after each ligand injection. Sequential dilutions of each scFv in range 0.375–12 μM were prepared in 50 mM sodium phosphate, pH = 7.4, on a 96-well plate. For binding of scFv to the peptide 50 mM sodium phosphate was used as running buffer and 50 mM BASE with 0.5 M NaCl was applied for regeneration of interaction surface.

## Results

Disulfide-independent folding of scFv may depend on multiple factors including the thermodynamic and proteolytic stability of the V_L_ and V_H_ domains in the absence of disulfide bonds. This includes contributions from the framework and from the CDRs, and also the interaction between the domains.

To examine the contribution of the framework and the CDRs to disulfide independent folding we chose four scFv from our previous study all of which could be assayed for binding activity. Trastuzumab and natalizumab were chosen as the only examples we have found experimentally to date to show efficient disulfide-independent folding in the cytoplasm of *E*. *coli* in deep well plates. To represent scFv that exhibited more disulfide-dependent folding we chose an IgA_1_ scFv as i) it showed good expression yields in the presence of CyDisCo [27]; ii) a crystal structure of Fab is available (3M8O, [35]); iii) the nature of the V_L_-V_H_ interface is very different from the other scFv we have examined using CyDisCo, with a strong electrostatic component; iv) to our knowledge CDRs swaps between IgG_1_/IgG_4_ and IgA derived scFv have not been previously reported. The other more disulfide-dependent folding scFv we chose was Maa48, an IgG_1_ which showed low yields using CyDisCo with an interest in determining why the low yields arose for the CyDisCo system development.

### Yields of scFv

**Fig 1:**
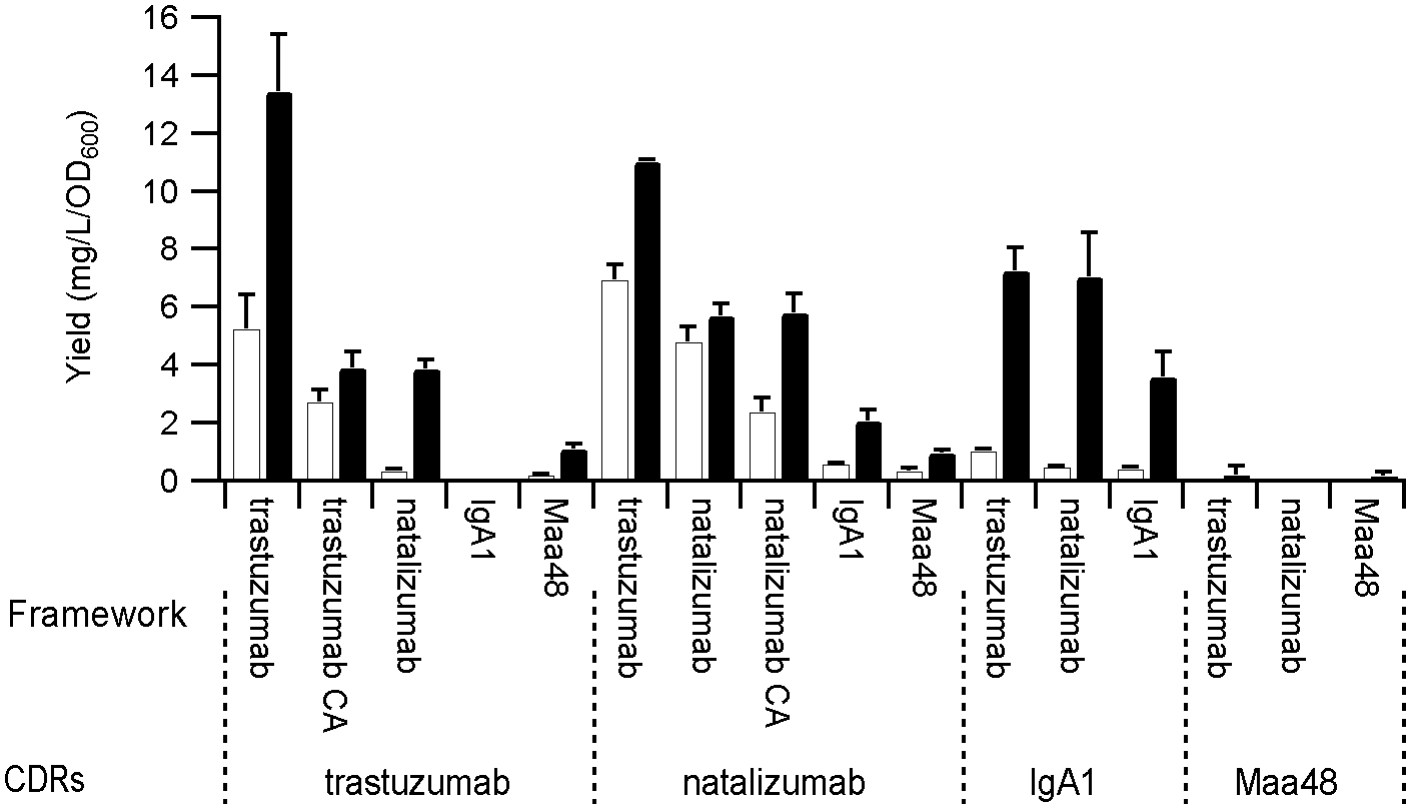
Comparison of the yields of purified scFv -/+ CyDisCo. Yields in mg/L/OD_600_ of scFv expressed with CyDisCo (black bars) or without (white bars). Average OD_600_ of the cultures was 22 ± 3 for those containing CyDisCo and 27 ± 3 for cultures without CyDisCo. Error bars show standard deviation, n = 3–4. scFv frameworks which have all four cysteines mutated to alanine are indicated as CA.

The four parental scFv were expressed in the cytoplasm of *E*. *coli* in the presence and absence of the CyDisCo system for disulfide bond formation. As previously, trastuzumab and natalizumab scFv showed disulfide-independent folding while the IgA_1_ and Maa48 scFv showed strongly disulfide-dependent folding (Fig 1). To confirm efficient disulfide-independent folding of trastuzumab and natalizumab scFv two independent methods were employed. Firstly, mass spectrometry confirmed the presence of disulfide bonds in purified proteins produced using CyDisCo and their absence in scFv produced without CyDisCo. Secondly, the four cysteine residues in trastuzumab and natalizumab scFv were mutated to alanine residues (denoted CA) and the mutant proteins were expressed and purified from the cytoplasm of *E*. *coli* in good yields (Fig 1). This is in contrast with previous reports on the mutated trastuzumab scFv produced in the periplasm forming inclusion bodies, but consistent with its *in vitro* refolding being disulfide-independent [7].

The CDRs from the strongly disulfide-independent scFv were transferred to the more disulfide-dependent frameworks and *vice versa* creating eight new hybrid scFv to examine whether the disulfide-dependence originated from the framework or from the CDRs. In addition, the CDRs from the two disulfide-independent scFv were swapped with the other disulfide-independent framework. The ten hybrid scFv were then expressed in the cytoplasm of *E*. *coli* in the presence and absence of the CyDisCo system. Two of the hybrids failed to produce any soluble protein under any condition tested, the natalizumab framework—Maa48 CDR hybrid forming inclusion bodies and the IgA_1_ framework—trastuzumab CDR hybrid showing proteolytic degradation. Of the remaining eight hybrid scFv all demonstrated folding in the presence of CyDisCo and seven in the absence of oxidative folding catalysts (Fig 1).

**Fig 2:**
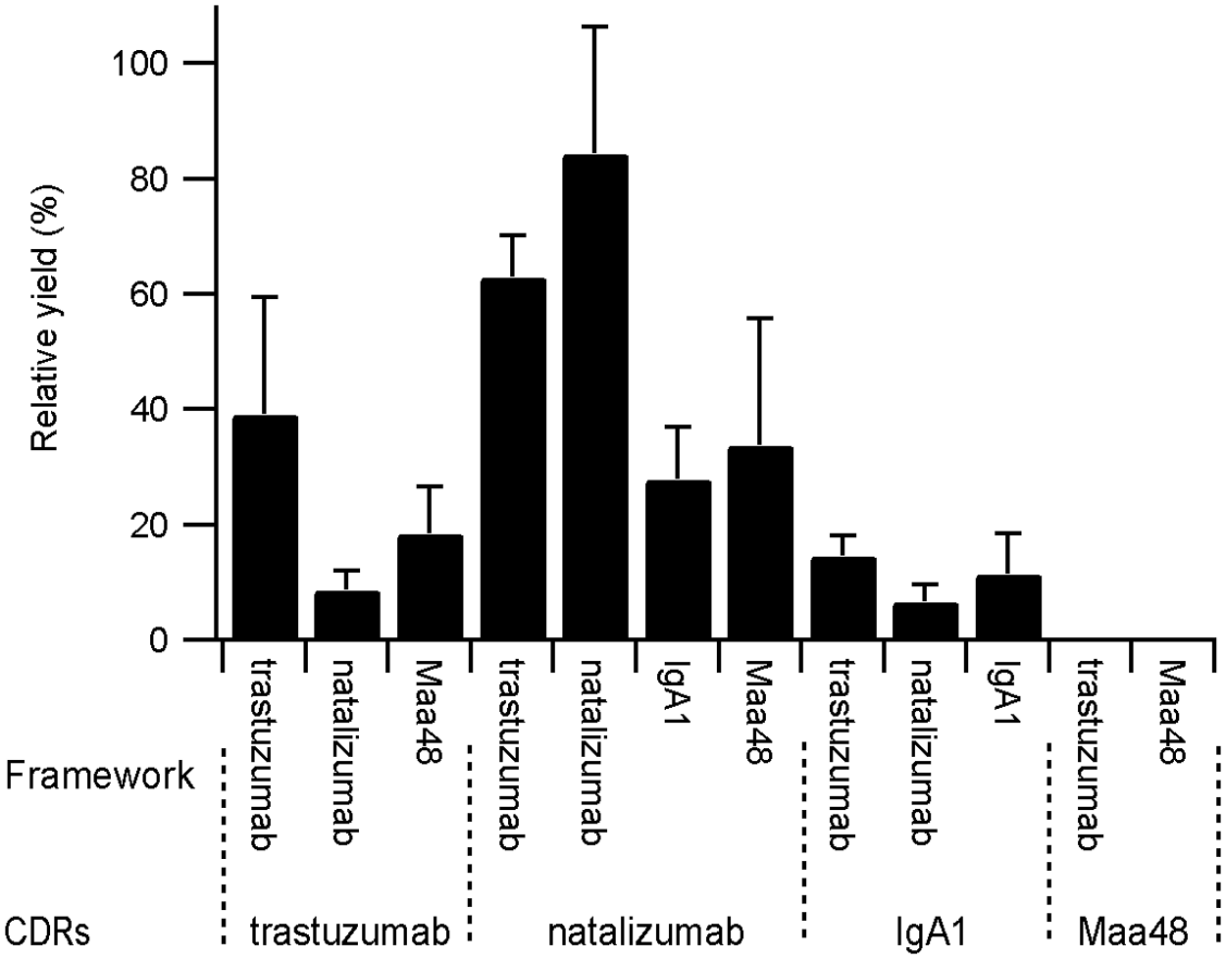
Relative disulfide-dependence of folding. Relative yield of scFv expressed without CyDisCo compared to the yield from expression with CyDisCo, n = 3–4.

For all constructs that could be produced in a soluble format there was a clear correlation in the yield obtained for any CDR set dependent on the framework used, with trastuzumab > natalizumab > IgA_1_ > Maa48. Similarly when examining the relative yield in the absence and presence of CyDisCo there is a clear dependence on the CDRs with the order of CyDisCo-independence being natalizumab > trastuzumab > IgA_1_ > Maa48 (Fig 2). Taken together the data implies that both the framework and the CDRs contribute to the disulfide-dependence of folding, with the CDRs having the largest effect.

With the exception of the Maa48-framework-trastuzumab CDRs hybrid made in the absence of CyDisCo which was prone to aggregation, to confirm the folded state and binding activity of the scFv produced electrospray ionization mass spectrometry (ESI-MS), circular dichroism (CD) and binding assays were undertaken of the purified scFv.

### Molecular masses and oxidation state analysis

ESI-MS indicated that with six exceptions the twenty-three scFv analysed had the expected mass, showed no proteolysis and had the expected disulfide status for the production conditions (Table 2). The IgA1 parental scFv and four of the hybrid scFv, the natalizumab framework—trastuzumab CDRs, natalizumab framework—IgA_1_ CDRs, IgA_1_ framework—natalizumab CDRs, and Maa48 framework—natalizumab CDRs were prone to oxidation during purification and storage and the material produced without CyDisCo was predominantly oxidized when analysed. These hybrids were not included in any subsequent analysis on the effects of disulfide-bonds since the fully reduced material was not the majority in the purified state. Similarly the trastuzmab framework—IgA_1_ CDR hybrid showed some oxidation during purification and storage. The seventh unexpected result was that the trastuzumab framework—natalizumab CDR hybrid scFv showed batch to batch variation in the degree of disulfide bond formation with CyDisCo. This is the only scFv which we have observed this for, with >98% of all proteins tested to date using CyDisCo showing homogenous disulfide bond formation. For this hybrid a batch of protein in which the disulfide bonds were formed was used in subsequent analysis.

### Secondary structure analysis

**Fig 3:**
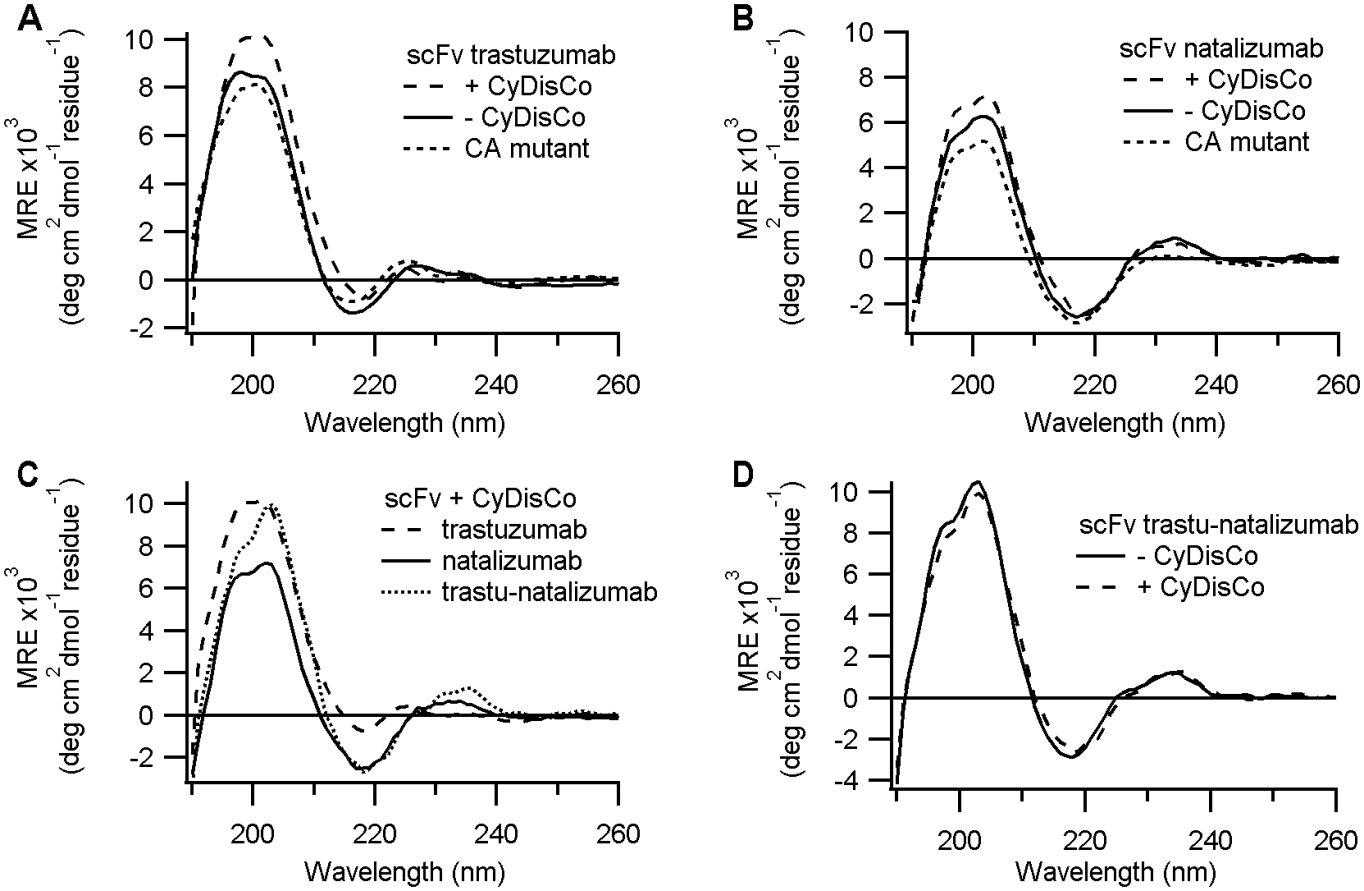
Far UV circular dichroism spectra of purified scFv. CD spectra of scFv that were found to be soluble in the absence of disulfide bonds and comparisons with the disulfide bonded counterpart. (A) scFv trastuzumab; (B) scFv natalizumab; (C) scFv trastuzumab and natalizumab and the trastuzumab framework—natalizumab CDR hybrid scFv all produced with CyDisCo; (D) trastuzumab framework—natalizumab CDR hybrid scFv. scFv frameworks which have all four cysteines mutated to alanine are indicated as CA.

CD analysis indicated that all purified scFv exhibited far UV CD spectra with a minima around 217 nm, consistent with having an IgG-fold, though framework and CDRs dependent differences were observed (Fig 3). Small differences in spectra, consistent with small differences in secondary structure was observed for trastuzumab with and without disulfide bonds and in mutant forms of trastuzumab lacking cysteines (Fig 3A), indicating a contribution of the disulfide bonds to secondary structure, while natalizumab showed a smaller contribution of disulfide bonds to secondary structure (Fig 3B). The differences in CD spectra between scFv appeared to reside primarily in the CDRs rather than in the framework (Fig 3C). While trastuzumab showed CD spectra that was disulfide-dependent (Fig 3A), the trastuzumab framework—natalizumab CDR hybrid demonstrated CD spectra that was disulfide-independent (Fig 3D) as per natalizumab scFv (Fig 3B).

### Binding activity analysis

**Fig 4:**
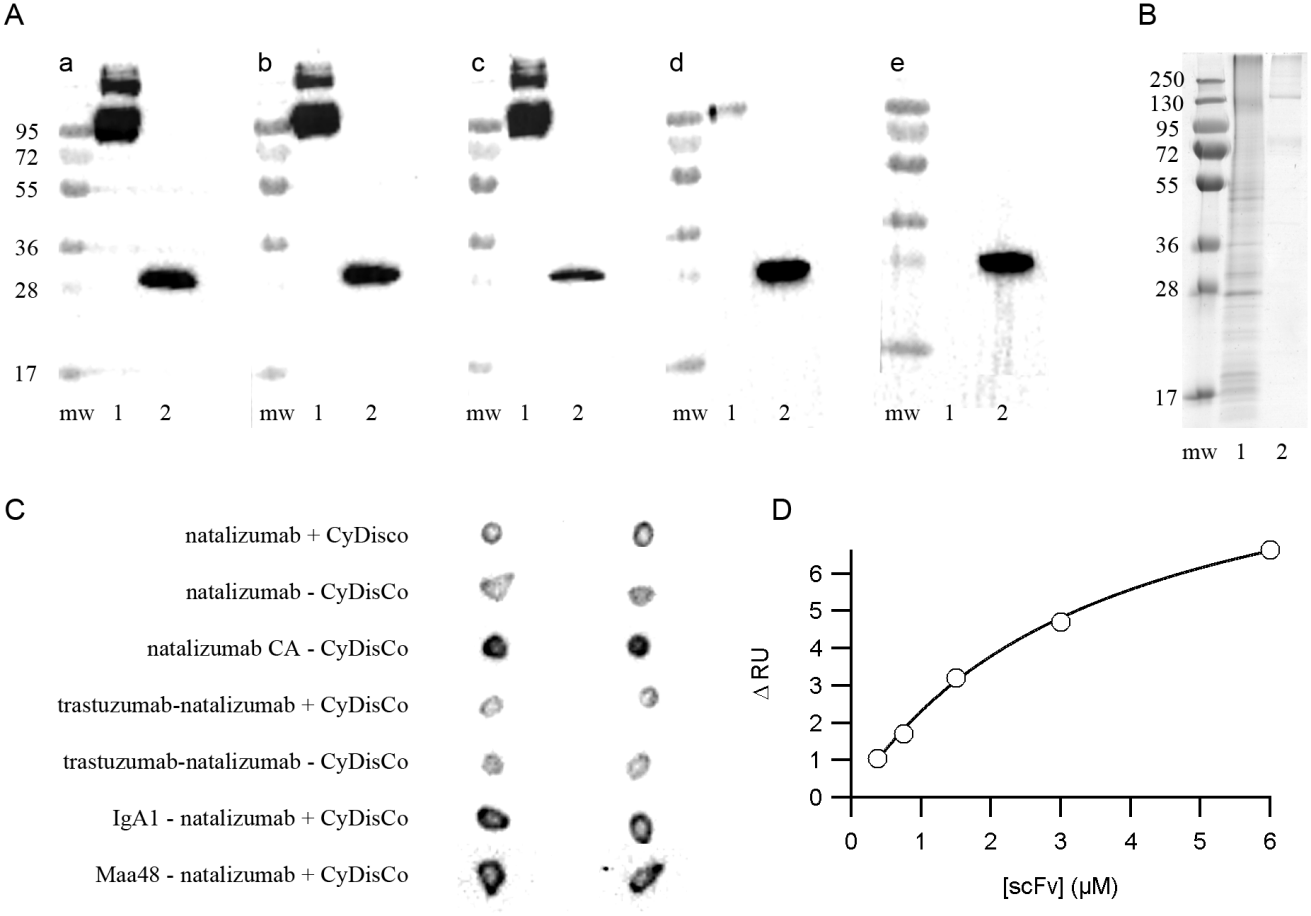
Binding activity of purified scFv. (A) western blot showing lane 1—binding of scFv with trastuzumab CDRs to the extracellular part of HER2 and lane 2—control sample showing binding of scFv with trastuzumab CDRs to the anti-His-tag secondary antibody; (a) trastuzumab + CyDisCo, (b) trastuzumab − CyDisCo, (c) trastuzumab CA − CyDisCo, (d) natalizumab framework-trastuzumab CDRs + CyDisCo, (e) Maa48 framework-trastuzumab CDRs + CyDisCo; (B) analysis of full length ITGA4 (purchased from 1. Abnova and 2. OriGene) on non-reducing SDS-PAGE; (C) dot blot of scFv containing natalizumab CDRs binding to full length ITGA4; (D) representative example of surface plasmon resonance (Biacore) analysis of binding of parental scFv IgA_1_ to β-tubulin peptide epitope.

That CDRs can be swapped between different frameworks while retaining binding activity is well documented in the literature [16,19,20]. The four scFv used as the basis for this study have very different ligands. While ELISA is widely used to examine antigen-antibody interactions, we were concerned that misleading results might be obtained due to differences in secondary antibody binding to the various primary scFv. To examine binding activity we used three different methods. For trastuzumab and hybrid scFv containing trastuzumab CDRs western blotting was used with a scFv control lane to examine differences in secondary antibody recognition. The results indicate that some differences in secondary antibody binding were observed (compare the intensity of bands in lane 2 between subpanels in Fig 4A) and that trastuzumab binding to its ligand HER2 is independent of disulfide bonds, consistent with previous results with this scFv [7], but that the trastuzumab CDRs in the natalizumab framework shows a reduced signal in western blotting while the trastuzumab CDRs in the Maa48 framework hybrid scFv shows no signal (Fig 4A). This alteration in signal intensity could arise either from differences in SDS-sensitivity or from altered affinity for the ligand in the western blot. Heterogeneity of the commercial HER2 (e.g. as seen Fig 4Aa lane 1) suggested a more quantitative assay format would be inappropriate from this sample.

To overcome the heterogeneity problem of commercially available HER2 we produced an 11.9 kDa HER2 fragment that contains the epitope to which trastuzumab binds (1N8Z; [33]). This fragment of HER2 contains six disulfide bonds, nevertheless it was successfully produced in the presence of CyDisCo in the cytoplasm of *E*. *coli*. Apparent homogeneity of the produced HER2 fragment was confirmed by SDS-PAGE analysis and mass spectrometry. Binding of scFv containing trastuzumab CDRs to the HER2 fragment was examined via Surface Plasmon Resonance (SPR). The binding trend (Table 3) is similar to results obtained via western blotting, with scFv trastuzumab binding with similar affinity independently of disulfide bond status and the natalizumab framework—trastuzumab scFv showing the second highest affinity. The Maa48 framework—natalizumab CDRs scFv showed circa 2.5x lower affinity than that of the parental scFv—in contrast with the results from western blotting (Fig 4Ae), in which no binding was detected for this hybrid scFv. This difference may arise either due to use of denaturated HER2 in western blot comparing with native state HER2 fragment used in SPR analysis or due to the indirect nature of western blotting.

**Table 3:** K_D_ determination for scFv with trastuzumab CDRs binding to HER2 fragment V529-P625.

scFv	CyDisCo	K_D_ (nM)
trastuzumab	+	96.8 ± 15
trastuzumab	-	103.5 ± 31
trastuzumab CA	-	88.5 ± 13
natalizumab-trastuzumab	+	147.1 ± 28
Maa48-trastuzumab	+	258.9 ± 65

The K_D_ for the scFv as determined by SPR are indicated along with whether the scFv were expressed with or without CyDisCo components. K_D_ values were determined from a standard curve with 5 concentration data points. K_D_ values shown are mean +/- standard deviation (n = 3). scFv frameworks which have all four cysteines mutated to alanine are indicated as CA.

While trastuzumab recognizes its ligand in western blotting, natalizumab recognizes a conformational epitope in its ligand ITG4A as seen in the crystal structure of the complex (PDB entry 4IRZ; [34]). As such it is unlikely to work in western blotting. Commercially available ITG4A from multiple sources showed significant heterogeneity on non-reducing SDS-PAGE (Fig 4B) and hence dot-blot rather than a more quantitative method was chosen to assay the activity of natalizumab and natalizumab CDRs containing hybrid scFv. A control with a fragment of ITG4A which includes only part of the natalizumab epitope was negative for all scFv tested (data not shown), while a scFv control dot blot showed that all were recognized by the secondary antibody (data not shown). Like trastuzumab scFv, natalizumab scFv demonstrated a disulfide-independent binding to its antigen (Fig 4C). All natalizumab CDRs containing hybrid constructs also showed binding to ITG4A (Fig 4C). Differences in signal intensity were observed, with several constructs giving stronger signals than the parental natalizumab scFv. This difference in signal intensity could arise from altered affinity for the ligand or from alterations in recognition by the secondary antibody though the scFv blotted control gave a strong signal in all cases. In an attempt to allow a more quantitative assay format to be used we tried to produce ITG4A using CyDisCo, but initial attempts were unsuccessful with the protein forming inclusion bodies (data not shown).

The chosen IgA_1_ scFv recognizes a linear epitope in β-tubulin making it ideal for studies determining both recognition of the epitope in the CDR swap hybrids as well as the affinity for the antigen. SPR was chosen to determine affinity to an immobilized β-tubulin peptide. All constructs with IgA_1_ CDRs demonstrated affinity to the peptide (Table 4 and representative example of parental scFv IgA_1_ binding to the antigen in Fig 4D) with the trastuzumab framework hybrid showing similar affinity as the IgA_1_ scFv, while the natalizumab framework hybrid showed circa six-fold lower affinity. The trastuzumab framework hybrid scFv had a circa two-fold higher affinity in the absence of disulfide bonds.

**Table 4:** K_D_ determination for scFv with IgA_1_ CDRs binding to β-tubulin peptide.

scFv	CyDisCo	K_D_ (μM)
IgA_1_-IgA_1_	+	3.56 ± 0.65
trastuzumab-IgA_1_	+	3.38 ± 0.36
trastuzumab-IgA_1_	-	1.45 ± 0.16
natalizumab-IgA_1_	+	21.3 ± 4.1

The K_D_ for the scFv as determined by SPR are indicated along with whether the scFv were expressed with or without CyDisCo components. K_D_ values were determined from a standard curve with 5 concentration data points. K_D_ values shown are mean +/- standard deviation (n = 3)

### Thermal stability analysis

**Fig 5:**
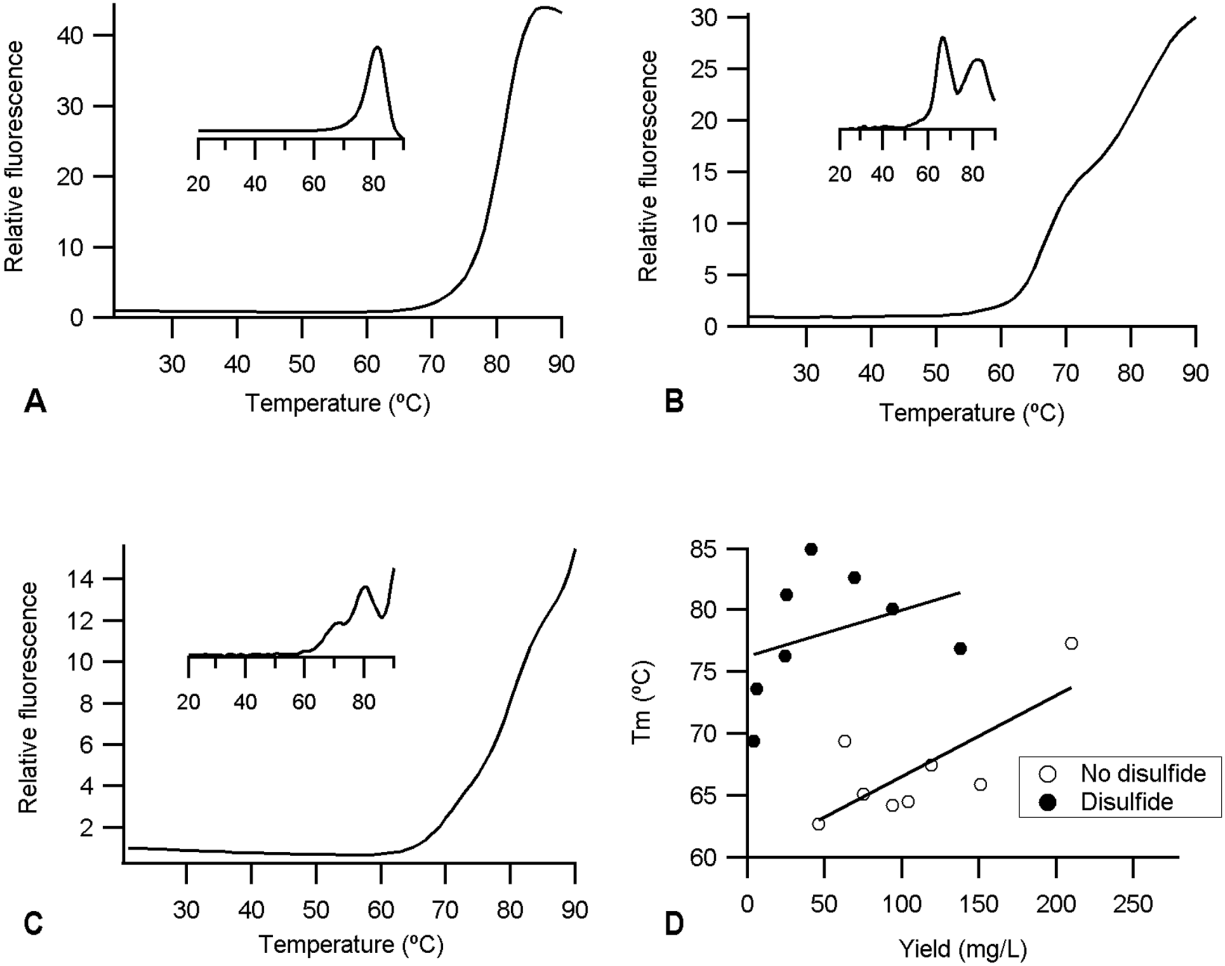
Thermal stability of the purified scFv that were expressed with CyDisCo. (A) natalizumab framework—trastuzumab CDRs hybrid scFv. (B) natalizumab scFv. (C) trastuzumab scFv. For panels A-C all scFv were made in the presence of CyDisCo. The insert panel in each case shows the derivative of the change in fluorescence signal. (D) Correlation between melting temperature (Tm) and the yield of purified scFv expressed with (●) or without (○) disulfide bonds. Only those scFv showing a single thermal denaturation step are plotted. Coefficient of correlation between Tm and the yield of scFv with disulfide bonds R^2^ = 0.12 and without disulfide bonds R^2^ = 0.56.

Most likely the disulfide-dependence/independence of folding of different scFv arises from differences in stability, with disulfide-independent scFv being more stable and hence being sufficiently stable to attain a native-like structure even in the absence of the structure stabilizing disulfide bond. To test this hypothesis thermofluor analysis was undertaken to determine the thermal stability of the purified scFv. The majority of the scFv showed either one or two thermal transitions—most easily seen in the derivative of the temperature dependence of the fluorescence. One step thermal denaturation, for example the natalizumab framework—trastuzumab CDR hybrid scFv (Fig 5A), represents either cooperative unfolding of the V_L_ and V_H_ domains or indicates that the thermal stability of the two domains is similar. A two step thermal denaturation, for example natalizumab scFv (Fig 5B), represents independent unfolding of the V_L_ and V_H_ domains and indicates that they have different thermal stabilities. Of the twenty scFv tested, sixteen showed one step denaturation and three showed two step denaturation, with all three having either the trastuzumab or natalizumab framework (Table 5). One scFv, the trastuzumab scFv made in the presence of CyDisCo, showed a three-step transition (Fig 5C). The third transition was seen as a small shoulder in the thermofluor trace. It could represent a mixed species e.g. a proportion of the scFv lacking one or more disulfide bonds. However, no indication of such a species was observed by ESI-MS and the mid-point temperature of the transition (71.3°C) is significantly higher than the single step transition observed for trastuzumab scFv made in the absence of CyDisCo (66.5°C). More probably this transition represents disassociation of the V_L_-V_H_ interface, with the concomitant exposure of a small hydrophobic area and hence increase in signal in the thermofluor.

**Table 5:** Thermal stability of the scFv.

scFv (framework-CDRs)	CyDisCo expression	Temp (°C)
trastuzumab-trastuzumab	+	71.3 ± 0.9
		79.8 ± 0.4
		>90
	-	66.5 ± 0.6
trastuzumab CA-trastuzumab	+	64 ± 0.4
	-	65.5 ± 1.6
natalizumab-trastuzumab	+	80.5 ± 0.5
Maa48-trastuzumab	+	78.6 ± 0.6
trastuzumab-natalizumab	+	80 ± 0
		>90
	-	77 ± 0
natalizumab-natalizumab	+	67 ± 0.3
		82.1 ± 0.8
	-	65 ± 1.1
natalizumab CA-natalizumab	+	69.7 ± 2.0
	-	70.3 ± 0.6
IgA_1_-natalizumab	+	84.3 ± 0.9
Maa48-natalizumab	+	80.3 ± 0.6
trastuzumab-IgA_1_	+	75 ± 0
		83.5 ± 0.5
	-	63.5 ± 0.6
natalizumab-IgA_1_	+	78.3 ± 0.4
IgA_1_-IgA_1_	+	83 ± 0
trastuzumab-Maa48	+	73.3 ± 0.6
Maa48-Maa48	+	70.3 ± 0.6

The mid-point temperature for thermal denaturation of the scFv as determined by thermofluor are indicated along with whether the scFv were expressed with or without CyDisCo components (n = 3–6). The scFv which were prone to *ex vivo* oxidation are not included. scFv frameworks which have all four cysteines mutated to alanine are indicated as CA.

Cross comparing the scFv which could be made with and without disulfide bonds, the presence of the disulfide bond clearly contributes significantly to the stability of the V_H_ and V_L_ domains, with the exception of one of the domains in natalizumab which is only stabilized by 2°C. The trastuzumab scFv with cysteines mutated to alanines was 1°C less stable than the disulfide free wild-type while the corresponding natalizumab mutant was 4°C more stable than the disulfide free wild-type protein.

While disulfide bonds contribute to the stability of all of the scFv tested there was no correlation between thermal stability of the proteins and the disulfide-dependent/independent folding with disulfide-dependent scFv having higher e.g. IgA_1_ or lower e.g. Maa48 thermal stability than the disulfide-independent natalizumab scFv. Furthermore only a weak correlation could be observed between the yield of protein obtained and the thermal stability of the protein (Fig 5D) further indicating that the high yield production of scFv is not directly linked to the stability of the folded protein. This is in contrast to previous results which found an excellent quantitative correlation between stability and yield [11].

## Discussion

The ability of scFv to attain a disulfide-independent native-like state is multi-factorial. Firstly, the thermodynamics must favor the same tertiary structure in the presence or absence of a disulfide bond. Usually disulfide bonds stabilize the native state and hence for disulfide-independent folding either the contribution of the disulfide bond to the stability of the protein must be small or the stability of the native state must be high such that when the stabilizing disulfide is removed a stable native-like state can still be achieved. Both of these possibilities were observed here. The thermal stability of one domain of natalizumab scFv showed a minor dependence on disulfide state, while both domains of trastuzumab scFv were significantly destabilized in the absence of disulfide bonds the disulfide-free state still had a melting temperature of circa 66°C.

We hypothesized that the framework would play a larger role in the disulfide-dependence/independence than the CDRs. This turned out not to be the case, with both the framework and the CDRs contributing significantly to the effect. Furthermore, within the limited set tested here, the CDRs appeared to play a greater role than the framework for disulfide-independence as well as for the structural dependence of the folded state on the disulfide. Conversely the framework appeared to play the major role in the yield of protein obtained.

In a simple model there would be expected to be a correlation between disulfide-dependence and stability of the native state, but no such simple correlation could be observed with the data collected here. Similarly there was only a weak correlation between the thermal stability of the scFv and the yield of purified protein. The two strongly disulfide-independent parental scFv showed independent unfolding of the V_L_ and V_H_ domains, in the disulfide bonded form, while the two more disulfide-dependent parental scFv showed a single thermal unfolding transition hinting at a role for domain interactions in disulfide-dependence, however this correlation did not extend to the hybrid scFv. A larger data set might clarify this.

The strong role of the CDRs in disulfide-independence is not easily explained. Of the 10 scFv we have published as having previously produced using CyDisCo [27] and the 49 we have not published, we have observed strong disulfide-independence for only two, the scFv derived from natalizumab and trastuzumab. The crystal structures of the V_L_ and V_H_ domains of 9 of these 52 antibodies are available, but these are most usually as Fab fragments and/or in complex with antigens rather than as unliganded scFv making a molecular level explanation problematic. Visual examination of the structures as well as jsPISA (Protein Interfaces, Surfaces and Assemblies—software) [38,39] analysis of the V_L_-V_H_ domain interfaces revealed no common features that natalizumab and trastuzumab share which the others do not. Similarly examination of the framework sequences of the 59 scFv produced to date using CyDisCo showed no sequence features which are shared by natalizumab and trastuzumab but which are not found in the others i.e. no features which would correlate directly with disulfide-dependency could be identified from the primary structure. A sequence alignment of the CDRs similarly showed no correlation, however the net charge (lysine plus arginine vs glutamic and aspartic acid) on the CDRs of natalizumab and trastuzumab is zero, a feature shared with only one other murine scFv we have produced. Since the CDRs from the V_L_ and V_H_ domains are juxtaposed in the folded structure a net negative or positive charge might result in electrostatic repulsion hindering folding and hence the attainment of the native or native-like state. However, this effect cannot be large as the IgA_1_ we used here has net +3 charges in the CDRs and yet when put into the natalizumab framework it shows significant CyDisCo-independent folding.

The poor correlation observed between yield and stability was unexpected. Multiple factors can effect yield, but the production system, inter-domain linker, expression conditions, plasmid backbone (including promoter, terminator, cloning sites used and ribosome binding site) and purification methodology used here were identical for all scFv. Most likely then the differences in yields arise either from differences in folding efficiency or from differences in proteolytic stability. For those scFv which could be made, the yield correlates strongly with the framework used, with trastuzumab > natalizumab > IgA_1_ > Maa48 for the subset used here, but the CDRs also play a strong role e.g. Maa48 yields appear to be poor both because of the framework and the CDRs. Separating the effects of proteolysis vs folding is complex given their inter-dependence—proteolysis can occur in both the folded state and in folding intermediates. Examination of the sequences and available structures for the scFv produced using CyDisCo showed no obvious reasons for predicted differences in proteolytic stability or folding. For example there was no correlation between yield and the number or position of proline residues, prolyl-isomerization being a rate limiting step in protein folding, including in antibody fragment folding [4,26].

## Conclusions

Here we studied cytoplasmic production of scFv in *E*. *coli* in order to elucidate the disulfide-dependency of folding to aid design and production of robust intrabodies. Disulfide-independent folding in the reducing environment of cytoplasm was found to depend not only on the framework region, but also on the CDRs. In particular our results demonstrate that grafting CDRs from a more disulfide-independent scFv to the framework of a more disulfide-dependent scFv may create a hybrid scFv that is more disulfide-independent (e.g. IgA1 framework—natalizumab CDRs).

The protein yield was clearly affected by framework and CDRs used, with hybrid scFv based on trastuzumab framework being expressed in the highest yields in presence of CyDisCo and scFv containing natalizumab CDRs showing increased relative yields in the absence of oxidative folding catalysts. However, there was only a weak correlation between the thermal stability and the yield of soluble scFv. CDRs also seem to have more relevance for thermal stability then frameworks, with hybrid scFv with grafted CDRs of natalizumab or trastuzumab having higher denaturation temperature then the paternal scFv with the same framework.

A study of a much larger data set is required to elucidate all of the mechanistic principals underlying disulfide-independent folding of scFv, but clearly CDRs play a critical role and there is no simple correlation between stability and disulfide-independence.
